# ﻿Three new species of the wolf spider genus *Sinartoria* Wang, Framenau & Zhang, 2021 (Araneae, Lycosidae) from southern China

**DOI:** 10.3897/zookeys.1244.149186

**Published:** 2025-07-10

**Authors:** Jin-Zhen Lu, Zhi-Sheng Zhang, Lu-Yu Wang

**Affiliations:** 1 Key Laboratory of Eco-environments in Three Gorges Reservoir Region (Ministry of Education), School of Life Science, Southwest University, Chongqing 400715, China Southwest University Chongqing China

**Keywords:** Artoriinae, description, live coloration, morphology, taxonomy

## Abstract

Three new species of the wolf spider genus *Sinartoria* Wang, Framenau & Zhang, 2021 are described from China: *Sinartoriadui***sp. nov.** (♂♀, Guangxi), *S.maolan***sp. nov.** (♀, Guizhou) and *S.nanling***sp. nov.** (♀, Guangdong). Morphological descriptions, photographs, illustrations of copulatory organs and a distribution map are provided. Additionally, photographs of living species of this genus are provided for the first time.

## ﻿Introduction

The subfamily Artoriinae Framenau, 2007 was proposed based on the presence of a basoembolic apophysis in the male pedipalp, which is unique within Lycosidae (Framenau 2007). Li et al. (2012) first documented *Artoriaparvula* Thorell, 1877 in China and transferred *Hygrolycosaligulacea* (Qu, Peng & Yin, 2009) to the genus *Artoria* Wang, Framenau & Zhang, 2021. Wang et al. (2019) first recorded the genus *Artoria* in Malaysia and described a new species: *A.weiweii*. Wang et al. (2021) further studied the species of the subfamily Artoriinae in China, described a new genus, *Sinartoria*, and three new species (*S.damingshanensis*, *S.zhuangia*, and *A.hamata*); Wang and colleagues subsequently described the new species *A.subhamata* (Wang and Zhang 2022). Wang et al. (2024) first recorded the species *S.hamata* of the subfamily Artoriinae in Vietnam. Fomichev et al. (2024) described a new genus *Pamirosa*, and a new species *P.kudratbekovi*, of the subfamily Artoriinae, distributed in the Pamir Plateau (Tajikistan). Recently, three new species of this genus were described: *P.alaica*, *P.archalturica*, and *P.transalaica* (Fomichev and Omelko 2025). Thus far, there are a total of 13 species in three genera of Artoriinae in Asia, distributed across China, Indonesia, Kyrgyzstan, Malaysia, New Guinea, Philippines, Tajikistan and Vietnam (WSC 2025).

The wolf spider genus *Sinartoria* Wang, Framenau & Zhang, 2021 is a small group comprising three species: *Sinartoriadamingshanensis* Wang, Framenau & Zhang, 2021 (China, Guangxi), *Sinartoriahamata* Wang & Li, 2024 (Vietnam, Cat Ba National Park) and *Sinartoriazhuangia* Wang, Framenau & Zhang, 2021 (China, Guangxi) (Wang et al. 2024; WSC 2025).

Here, we describe three new species of *Sinartoria* from southern China. Additionally, we provide photographs of live *Sinartoria* specimens for the first time.

## ﻿Material and methods

All specimens are preserved in 75% ethanol. The specimens were examined, measured, and photographed using a Leica M205A stereomicroscope equipped with a Leica DFC450 camera and LAS software (ver. 4.6). The left male pedipalp was used for photography. Epigynes were cleared by immersion in pancreatin solution before examination and photography following Álvarez-Padilla and Hormiga (2007). Leg measurements are shown as total length (femur, patella and tibia, metatarsus, tarsus). All measurements are in millimeters. All specimens examined are deposited in the spider collection at the School of Life Sciences, Southwest University, Chongqing, China (**SWUC**). Terminology follows Wang et al. (2021).

Abbreviations used in the text and figures: **AA**–anterior arm of median apophysis; **ALE**–anterior lateral eye; **AME**–anterior median eye; **BEA**–basoembolic apophysis; **C**–conductor; **CO**–copulatory opening; **Em**–embolus; **FD**–fertilization duct; **HS**–head of spermatheca; **MA**–median apophysis; **PLE**–posterior lateral eye; **PME**–posterior median eye; **PA**–posterior arm of median apophysis; **RA**–retrolateral arm of median apophysis; **Se**–septum; **SS**–stalk of spermatheca; **St**–subtegulum; **TA**–terminal apophysis; **Te**–tegulum.

## ﻿Taxonomy

### ﻿Family Lycosidae Sundevall, 1833 (狼蛛科)

**Subfamily Artoriinae Framenau, 2007** (阿狼蛛亚科)

#### ﻿Genus Sinartoria

Taxon classificationAnimaliaAraneaeLycosidae

Wang, Framenau & Zhang, 2021

1569CB66-537B-53F0-AA38-A42030B0DF6D

##### Type species.

*Sinartoriadamingshanensis* Wang, Framenau & Zhang, 2021.

##### Diagnosis.

Males of this genus is similar to *Lobizon* Piacentini & Grismado, 2009 in having a long embolus resting in a groove of a broad terminal apophysis, but can be distinguished by several key characters: (1) a strongly sclerotized basoembolic apophysis (vs. weakly sclerotized and lamellar in *Lobizon*); (2) both membranous terminal apophysis 1 (TA1) and sclerotized, groove-like terminal apophysis 2 (TA2) are present (vs. TA2 only in *Lobizon*); and (3) a median apophysis with three arms (AA, RA, PA). Females can be recognized by a spermatheca with compact stalk and small protruding head (vs. stalk sinuous in *Lobizon*) (Wang et al. 2021; Wang et al. 2024).

##### Description.

Carapace yellowish brown. Eye region black with white setae. Chelicerae with 3 promarginal and 2 retromarginal teeth. Sternum yellow brown, heart-shaped. Legs with brown annulations. Opisthosoma oval, dorsum showing lanceolate cardiac mark anteriorly and black posterior patterning; males with white patch near anal tubercle. Ventral surface yellow with short spinnerets (Wang et al. 2021).

##### Composition.

Three new species: *S.dui* sp. nov., *S.maolan* sp. nov. and *S.nanling* sp. nov. *Sinartoriadamingshanensis* Wang, Framenau & Zhang, 2021, *S.hamata* Wang & Li, 2024 and *S.zhuangia* Wang, Framenau & Zhang, 2021.

##### Distribution.

China (Guangdong, Guangxi, Guizhou) and Vietnam.

#### ﻿ Sinartoria dui sp. nov.

Taxon classificationAnimaliaAraneaeLycosidae

C7EAF53D-FFF8-50D3-9734-DBEAC45D7D04

https://zoobank.org/D63658EE-4A60-4B12-8EF3-03EB0BF34E16

**Figure 1.**
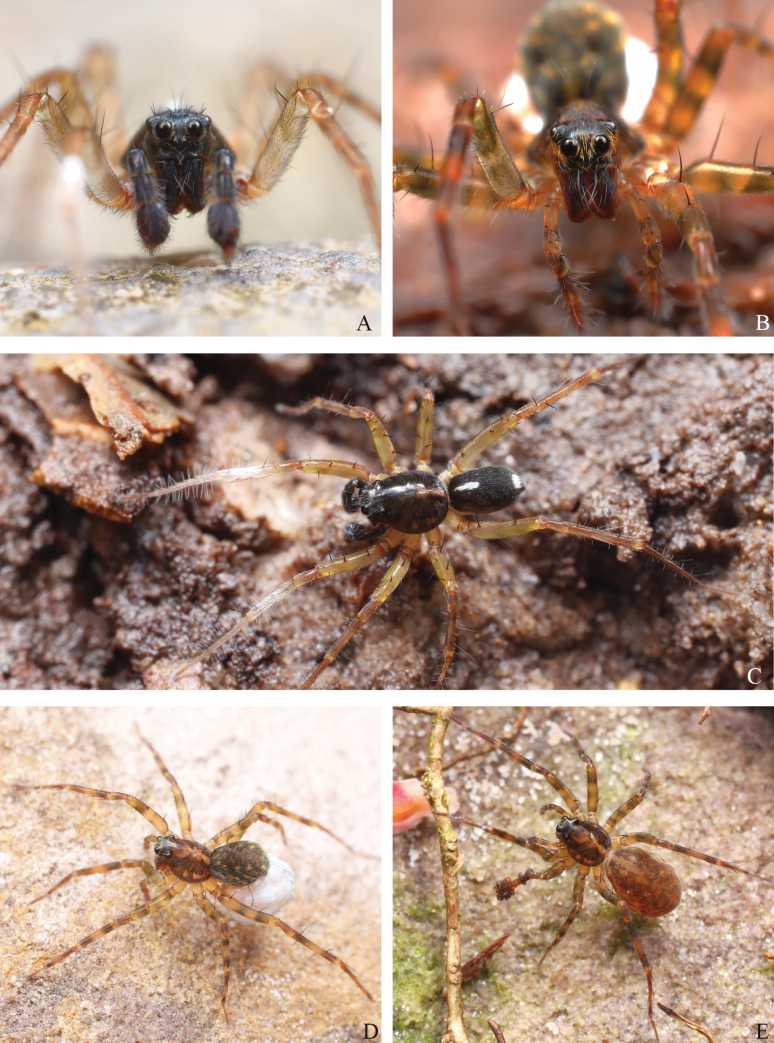
Photos of living *Sinartoriadui* sp. nov. **A, C.** Male; **B, D, E.** Female (photos taken by Qian-Le Lu).

**Figure 2.**
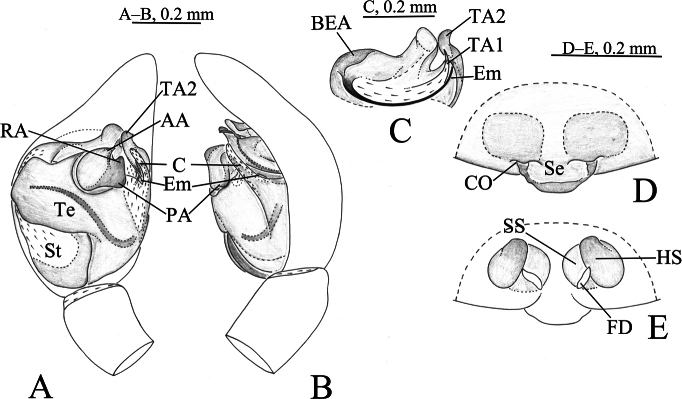
*Sinartoriadui* sp. nov. **A–C.** Holotype male; **D, E.** Paratype female; **A.** Left male palp, ventral view; **B.** Same, prolateral view; **C.** Embolic division, ventral view; **D.** Epigyne, ventral view; **E.** Vulva, dorsal view. Abbreviations: **AA**–anterior arm of median apophysis; **BEA**–basoembolic apophysis; **C**–conductor; **CO**–copulatory opening; **Em**–embolus; **FD**–fertilization duct; **HS**–head of spermatheca; **PA**–posterior arm of median apophysis; **RA**–retrolateral arm of median apophysis; **Se**–septum; **SS**–stalk of spermatheca; **St**–subtegulum; **TA1**–terminal apophysis 1; **TA2**–terminal apophysis 2;**Te**–tegulum.

**Figure 3.**
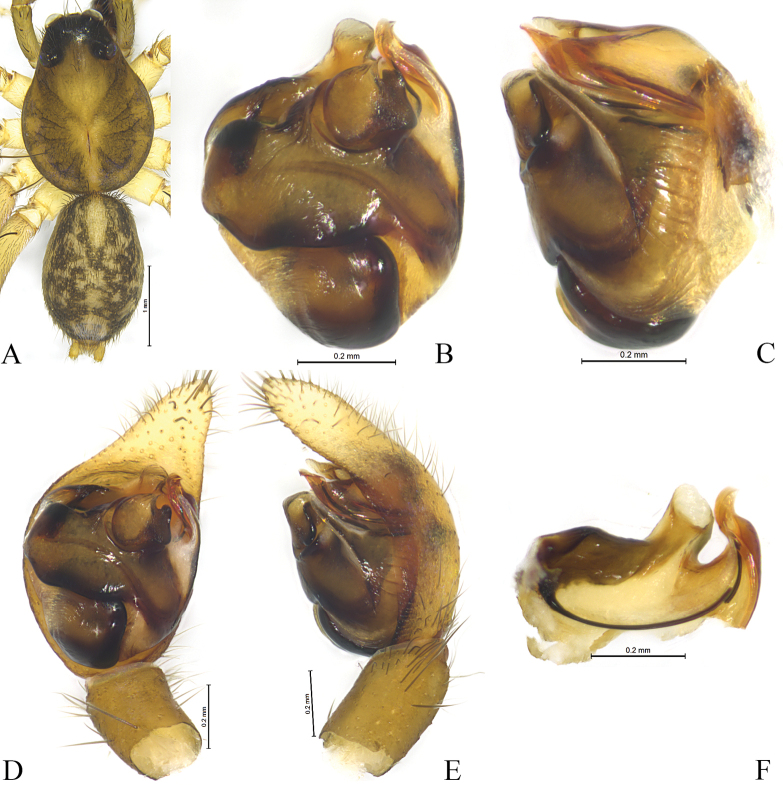
*Sinartoriadui* sp. nov. **A–F.** Holotype male; **A.** Male habitus, dorsal view; **B.** Bulb, ventral view; **C.** Same, prolateral view; **D.** Left male palp, ventral view; **E.** Same, retrolateral view; **F.** Embolic division, ventral view.

**Figure 4.**
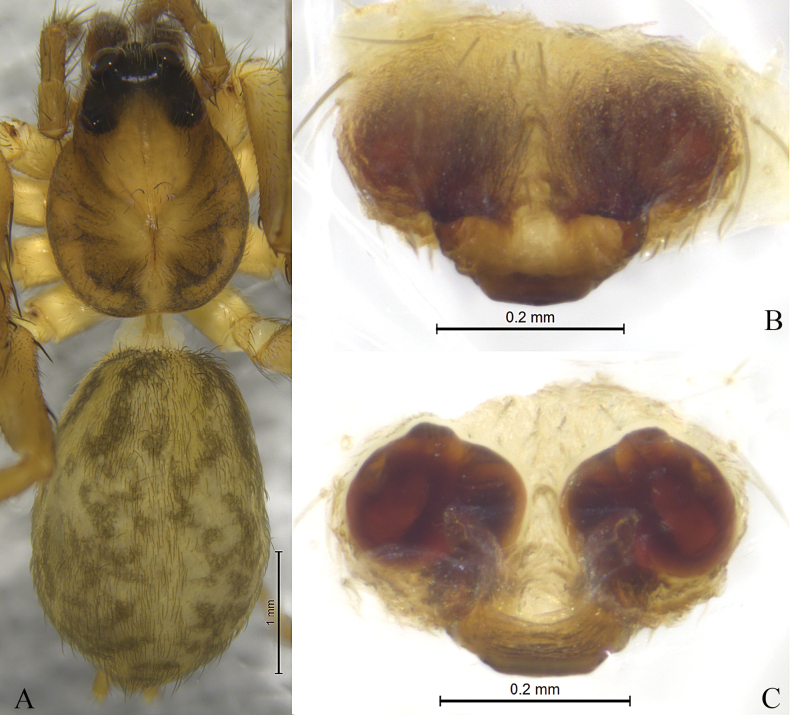
*Sinartoriadui* sp. nov. **A–C.** Paratype female; **A.** Female habitus, dorsal view; **B.** Epigyne, ventral view; **C.** Vulva, dorsal view.

Figs 1 , 2 , 3 , 4 (杜氏华阿狼蛛)

##### Type material.

***Holotype*** • ♂ (SWUC-T-LY-22-01), China, Guangxi, Laibin City, Jinxiu Co. 26 April 2023, W.Q. Zhao leg. (SWUC). ***Paratypes***: • 4♂3♀ (SWUC-T-LY-22-02 to 08), same data as holotype (SWUC) • 3♂3♀ (SWUC-T-LY-22-09 to 14), China, Guangxi, Jinxiu Co., Silverwood Forest park, 24°10'9"N, 110°14'38"E, elev. 1144 m, 26 April 2024, Q.L. Lu, C.C. Du and X.Y. Feng leg.

##### Etymology.

The specific name is derived from the family name of Dr Du Congcong of Guangxi Normal University, acknowledging his assistance in collecting specimens in Guangxi.

##### Diagnosis.

*Sinartoriadui* sp. nov. resembles *S.damingshanensis* Wang, Framenau & Zhang, 2021 in having a similar flamed-shaped terminal apophysis and three arms on the median apophysis in the male pedipalp and a large septum in the female epigyne (Figs 2A–D, 3B–F, 4B; Wang et al. 2021: 573, figs 1A–E, 2D–I). It can be differentiated from the latter by the lack of teeth on the anterior arm of median apophysis (Figs 2A, B, 3F; vs. with several teeth), a sheet-like retrolateral arm of median apophysis (Figs 2A, B, 3B–E; vs. knife-like); the trapezoid-shaped septum of the epigyne (Figs 2D, 4B; vs. somewhat triangular) and the proximally rod-like fertilization ducts (Figs 2E, 4C; vs. fertilization ducts crescent-shaped).

##### Description.

**Male** (holotype, Figs 1A, C, 3A) total length 4.16. Carapace 2.26 long, 1.68 wide; opisthosoma 1.93 long, 1.28 wide. Eye sizes and interdistances: AME 0.08, ALE 0.07, PME0.29, PLE 0.24; AME–AME 0.08, AME–ALE 0.11, PME–PME 0.23, PME–PLE 0.27. Clypeus height 0.07. Chelicerae yellow brown. Labium yellowish brown. Endites yellowish brown. Sternum black coarse long setae. Legs yellow brown, with brown pigmentation. Tibia with white setae, metatarsus and tarsus with notably long laterally protruding setae. Leg measurements: I 6.33 (1.78, 2.04, 1.64, 0.87); II 5.73 (1.58, 1.80, 1.51, 0.84); III 5.54 (1.47, 1.68, 1.57, 0.82); IV 8.09 (2.15, 2.48, 2.41, 1.05). Leg formula: 4123. Opisthosoma oval, dark brown dorsally, white spots on the front and end of the back.

***Pedipalp*** (Figs 2A–C, 3B–F). Median apophysis with three arms (AA, RA and PA) protruded, AA triangular, RA sheet-shaped, and PA finger-shaped. Embolus long and slender, anterior part rests in a long groove of a large and complex terminal apophysis. Terminal apophysis with two arms, one of them sclerotized and groove-like, the other one membranous.

**Female** (paratype, Figs 1B, D, E, 4A) total length 5.21. Carapace 2.19 long, 1.65 wide; opisthosoma 2.80 long, 1.93 wide. Eye sizes and interdistances: AME 0.09, ALE 0.08, PME 0.31, PLE 0.23; AME–AME 0.08, AME–ALE 0.10, PME–PME 0.24, PME–PLE 0.29. Clypeus height 0.07. Legs yellow brown, with brown pigmentation. Leg measurements: I 6.81 (1.97, 2.44, 1.45, 0.95); II 6.12 (1.77, 2.05, 1.46, 0.84); III 6.06 (1.71, 1.88, 1.62, 0.85); IV 8.76 (2.24, 2.83, 2.54, 1.15). Leg formula: 4123. Opisthosoma yellow brown dorsally, with black pattern. Ventral yellow brown.

***Epigyne*** (Figs 2D, E, 4B, C). Copulatory openings arc-shaped. Septum broad, trapezoid, narrow posteriorly. Spermathecal stalks slightly sclerotized, O-shaped. Spermathecal heads small with round end. Fertilization ducts small, with their length approximating the width of the spermathecal heads.

##### Habitat.

Beneath the humus stratum in deciduous broadleaf forests.

##### Distribution.

Known only from the type locality, Guangxi, China (Fig. 9).

#### ﻿ Sinartoria maolan sp. nov.

Taxon classificationAnimaliaAraneaeLycosidae

87C8A93C-8D5F-5A34-B147-0406A41C38D6

https://zoobank.org/4732AAC4-0EAF-4399-B980-030CF8B7EA46

Figs 5 , 6 (茂兰华阿狼蛛)

##### Type material.

***Holotype*** • ♀ (SWUC-T-LY-23-01), China, Guizhou, Libo Co. Maolan National Nature Reserve, Bizuo Town, 25°16'59"N, 108°03'18"E, elev. 587 m, 28 April 2017, R.X. Jiang leg.

##### Etymology.

The specific name is derived from the type locality; a noun in apposition.

##### Diagnosis.

**Figure 5.**
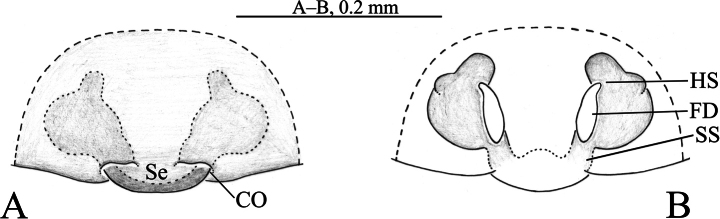
*Sinartoriamaolan* sp. nov. **A, B.** Holotype female; **A.** Epigyne, ventral view; **B.** Vulva, dorsal view. Abbreviations: **CO**–copulatory opening; **FD**–fertilization duct; **HS**–head of spermatheca; **Se**–septum; **SS**–stalk of spermatheca.

**Figure 6.**
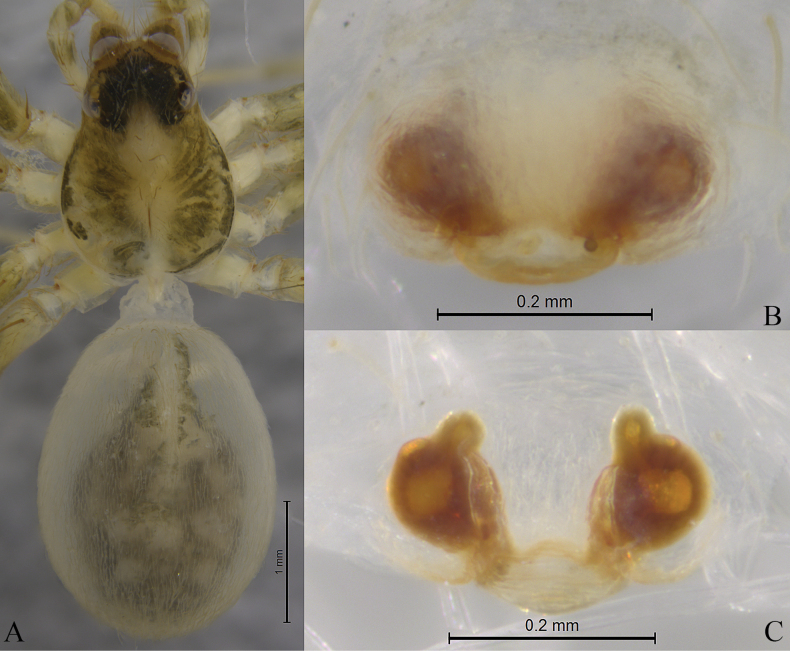
*Sinartoriamaolan* sp. nov. **A–C.** Holotype female; **A.** Female habitus, dorsal view; **B.** Epigyne, ventral view; **C.** Vulva, dorsal view.

**Figure 7.**
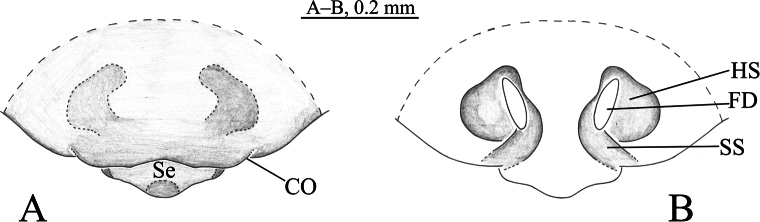
*Sinartoriananling* sp. nov. **A, B.** Holotype female; **A.** Epigyne, ventral view; **B.** Vulva, dorsal view. Abbreviations: **CO**–copulatory opening; **FD**–fertilization duct; **HS**–head of spermatheca; **Se**–septum; **SS**–stalk of spermatheca.

**Figure 8.**
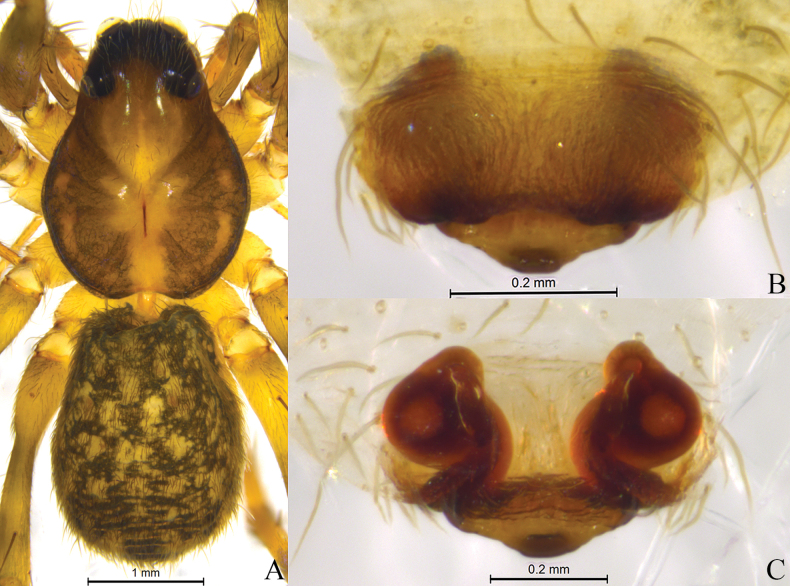
*Sinartoriananling* sp. nov. **A–C.** Holotype female; **A.** Female habitus, dorsal view; **B.** Epigyne, ventral view; **C.** Vulva, dorsal view.

Female of *Sinartoriamaolan* sp. nov. resembles *S.nanling* sp. nov. in having the small septum and spermathecal head subglobular (Figs 5A, B, 6B, C; Figs 7A, B, 8B, C), but can be differentiated from the latter by short spermathecal stalks, connected at the base (Figs 5B, 6C; vs. long, unconnected at the base) and proximally eggplant-shaped fertilization ducts (Figs 5B, 6C; vs. elliptical).

##### Description.

**Female** (holotype, Fig. 6A) total length 4.88. Carapace 1.92 long, 1.40 wide; opisthosoma 2.66 long, 1.96 wide. Eye sizes and interdistances: AME 0.07, ALE 0.08, PME 0.30, PLE 0.22; AME–AME 0.07, AME–ALE 0.10, PME–PME 0.21, PME–PLE 0.25. Clypeus height 0.04. Chelicerae yellow brown. Labium and endites yellow brown, longer than wide. Sternum yellow brown, shield-shaped, with long brown setae. Leg measurements: I 5.39 (1.56, 1.89, 1.19, 0.75); II 4.93 (1.39, 1.69, 1.09, 0.75); III 4.72 (1.33, 1.45, 1.19, 0.75); IV 6.78 (1.93, 2.19, 1.77, 0.89). Leg formula: 4123. Opisthosoma yellow brown dorsally, ventral side yellowish brown.

***Epigyne*** (Figs 5, 6B, C). Septum extends posteriorly. Spermathecal stalks short, connected at the base. Fertilization ducts large, proximally eggplant-shaped.

**Male.** Unknown.

##### Habitat.

Beneath the humus stratum in deciduous broadleaf forests.

##### Distribution.

**Figure 9.**
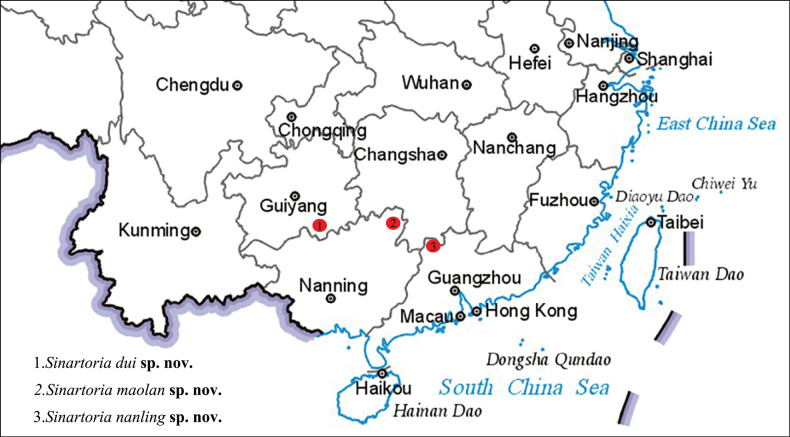
Distribution records of *Sinartoriadui* sp. nov., *Sinartoriamaolan* sp. nov. and *Sinartoriamaolan* sp. nov. in this study.

Known only from the type locality, Guizhou, China (Fig. 9).

#### ﻿ Sinartoria nanling sp. nov.

Taxon classificationAnimaliaAraneaeLycosidae

E9DA09EF-4BBA-52D6-9EAD-B97BAC3430FE

https://zoobank.org/2F65B577-B9E5-45B9-AD9E-C4FCB67A5785

Figs 7 , 8 (南岭华阿狼蛛)

##### Type material.

***Holotype*** • ♀ (SWUC-T-LY-24-01), China, Guangdong, Shaoguan City, Nanling Forest Park, July 2016, G.C. Zhou leg.

##### Etymology.

The specific name is derived from the type locality; a noun in apposition.

##### Diagnosis.

Female of *Sinartoriananling* sp. nov. resembles *S.maolan* sp. nov. in having the small septum and spermathecal head subglobular (Figs 7A, B, 8B, C; Figs 5A, B, 6B, C), but can be differentiated from the latter by long spermathecal stalks, unconnected at the base (Figs 7B, 8C; vs. short, connected at the base) and elliptical fertilization ducts (Figs 7B, 8C; vs. proximally eggplant-shaped).

##### Description.

**Female** holotype (Fig. 8A) total length 4.78. Carapace 2.45 long, 1.80 wide; opisthosoma 2.18 long, 1.73 wide. Eye sizes and interdistances: AME 0.08, ALE 0.06, PME 0.32, PLE 0.25; AME–AME 0.10, AME–ALE 0.09, PME–PME 0.23, PME–PLE 0.26. Clypeus height 0.09. Chelicerae yellow brown. Leg measurements: I 6.97 (1.97, 2.52, 1.59, 0.89); II 6.29 (1.78, 2.16, 1.55, 0.80); III 5.99 (1.65, 1.92, 1.62, 0.80); IV 8.80 (2.31, 2.77, 2.62, 1.10). Leg formula: 4123. Opisthosoma oval.

***Epigyne*** (Figs 7, 8B, C). Septum extends posteriorly. Spermathecal stalks long, unconnected at the base. Spermathecal subglobular. Fertilization ducts large, elliptical.

**Male.** Unknown.

##### Habitat.

Beneath the humus stratum in deciduous broadleaf forests.

##### Distribution.

Known only from the type locality, Guangdong, China (Fig. 9).

## ﻿Discussion

*Sinartoria* belongs to the subfamily Artoriinae, being very similar to the genus *Lobizon* Piacentini & Grismado, 2009. Lobizon consists of five species, all of which are found in Argentina. Considering the distribution of *Sinartoria* species, we speculate that Artoriinae may have originated from the ancient supercontinent of Gondwana (Piacentini and Grismado 2009; Li et al. 2012).

## Supplementary Material

XML Treatment for Sinartoria

XML Treatment for Sinartoria dui

XML Treatment for Sinartoria maolan

XML Treatment for Sinartoria nanling

## ﻿Additional information

### Use of AI

No use of AI was reported.

### Author ORCIDs

Jin-Zhen Lu https://orcid.org/0009-0006-1130-2954

Zhi-Sheng Zhang https://orcid.org/0000-0002-9304-1789

Lu-Yu Wang https://orcid.org/0000-0002-5250-3473

### Data availability

All of the data that support the findings of this study are available in the main text.

## References

[B1] Álvarez-Padilla F Hormiga G (2007) A protocol for digesting internal soft tissues and mounting spiders for scanning electron microscopy. The Journal of Arachnology 35(3): 538–542. 10.1636/Sh06-55.1

[B2] Fomichev AA Omelko MM (2025) Three new species of *Pamirosa* Fomichev, Omelko & Marusik, 2024 (Araneae: Lycosidae) from Kyrgyzstan, extending the known range of Artoriinae in Central Asia. Zootaxa 5618(2): 249–266. 10.11646/zootaxa.5618.2.4 40173463

[B3] Fomichev AA Omelko MM Marusik YM (2024) *Pamirosa* gen. nov., unexpected record of Artoriinae (Araneae, Lycosidae) from the rooftop of Pamir, Central Asia. Zoosystematics and Evolution 100(3): 1005–1015. 10.3897/zse.100.123331

[B4] Framenau VW (2007) Revision of the new Australian genus *Artoriopsis* in a new subfamily of wolf spiders, Artoriinae (Araneae: Lycosidae). Zootaxa 1391(1): 1–34. 10.11646/zootaxa.1391.1.1

[B5] Li ZX Framenau VW Zhang ZS (2012) First record of the wolf spider subfamily Arto­riinae and the genus *Artoria* from China (Araneae: Lycosidae). Zootaxa 3235(1): 35–44. 10.11646/zootaxa.3235.1.3

[B6] Piacentini LN Grismado CJ (2009) *Lobizon* and *Navira*, two new genera of wolf spiders from Argentina (Araneae: Lycosidae). Zootaxa 2195(1): 1–33. 10.11646/zootaxa.2195.1.1

[B7] Piacentini LN Ramírez MJ (2019) Hunting the wolf: A molecular phylogeny of the wolf spiders (Araneae, Lycosidae). Molecular Phylogenetics and Evolution 136: 227–240. 10.1016/j.ympev.2019.04.004 30953780

[B8] Wang LY Zhang ZS (2022) A new species of genus *Artoria* Thorell, 1877 from Sichuan, China (Araneae: Lycosidae). Acta Arachnologica Sinica 31(2): 109–112. 10.3969/j.issn.1005-9628.2022.02.008

[B9] Wang LY Zhang ZS Peng XJ (2019) First record of *Artoria* Thorell, 1877 (Araneae: Lycosidae) from Malaysia, with the description of a new species. Zootaxa 4657(2): 392–396. 10.11646/zootaxa.4657.2.12 31716793

[B10] Wang LY Framenau VW Zhang ZS (2021) A further study on the wolf spider subfamily Artoriinae from China (Araneae: Lycosidae). Zootaxa 4964(3): 571–584. 10.11646/zootaxa.4964.3.8 33903510

[B11] Wang LY Li SQ Pham DS (2024) The wolf spiders of three national parks in Northern Vietnam (Araneae: Lycosidae). Zootaxa 5537(2): 234–244. 10.11646/zootaxa.5537.2.4 39646341

[B12] WSC (2025) World Spider Catalog. Version 25.5. Natural History Museum Bern, online at http://wsc.nmbe.ch. 10.24436/2 [Accessed on 2025-2-8]

